# A workplace mindfulness training program may affect mindfulness, well-being, health literacy and work performance of upper-level ICT-managers: An exploratory study in times of the COVID-19 pandemic

**DOI:** 10.3389/fpsyg.2023.994959

**Published:** 2023-04-20

**Authors:** Kristina Schubin, Laura Seinsche, Holger Pfaff, Sabrina Zeike

**Affiliations:** ^1^Institute of Medical Sociology, Health Services Research and Rehabilitation Science, Faculty of Human Sciences, Faculty of Medicine and University Hospital Cologne, University of Cologne, Cologne, Germany; ^2^vivalue GmbH, Cologne, Germany

**Keywords:** mindfulness, manager, training, workplace, intervention, well-being, health literacy, work performance

## Abstract

**Introduction:**

Mindfulness-based interventions have gained more importance in workplace health promotion due to increased psychological distress in the digital era. Although managers in the information communication technology sector (ICT)-sector are at risk for lower mental health, few studies have evaluated the effects of workplace mindfulness trainings (WMT) on upper-level ICT-managers.

**Methods:**

By applying a mixed methods approach, the study aimed at exploring differences in upper-level ICT-managers’ mindfulness, well-being, health literacy and work performance at the beginning of a WMT (*t*0), immediately after (*t*1) and 3  months after (*t*2) a WMT. Thirteen groups of managers (*n* = 56) completed the training and three corresponding surveys consecutively from October 2019 to April 2021. Managers rated their mindfulness (MAAS), well-being (WHO-5), health literacy, and work performance (HPQ). During the COVID-19-pandemic the training switched from a live on-site mode to a hybrid mode and finally to a digital mode. Repeated measures ANOVAs and Bonferroni-adjusted *post hoc* analyses were used for data analysis. Open-ended responses were content analyzed.

**Results:**

We found significant differences in managers’ mindfulness [*F*(2.106) = 3.376, *p* = 0.038, η_p_^2^ = 0.06, *n* = 54], well-being [*F*(2.106) = 73.019, *p* < 0.001, η_p_^2^ = 0.17, *n* = 54], health literacy [*F*(2.108) = 9.067, *p* < 0.001, η_p_^2^ = 0.15, *n* = 55], and work performance [*F*(2.80) = 7.008, *p* = 0.002, η_p_^2^ = 0.15, *n* = 41] between *t*0 and *t*2. Significant differences between *t*0 and *t*1 were also found for well-being, health literacy and work performance, but not for mindfulness. Qualitative findings demonstrated positive training effects, barriers and facilitators to daily application of mindfulness practice.

**Discussion:**

The results suggest that compared to the beginning of the WMT, the post and follow-up measurements showed outcome improvements. The workplace mindfulness training may thus be a promising program to facilitate mental health and working capabilities among upper-level ICT-managers. Contextual workplace factors need to be considered to sustain long-term mindfulness practice of managers.

## 1. Introduction

As the working world is changing rapidly, managers have to operate in workplace settings that are becoming increasingly volatile, uncertain, complex, and ambiguous (Rodriguez and Rodriguez, 2015). Even before the COVID-19 pandemic, managers were confronted with (techno-)stress (Harms et al., 2017; Stadin et al., 2021), high workload (Eurofound, 2017), and with leading the so-called digital transition in companies (Westerman et al., 2015). Such work-related stress factors pose a risk for both the physical and mental health (Sohail and Rehmann, 2015; Hirschle and Gondim, 2020). This constitutes a call for action: As the workplace is an important setting to promote health among a broad worker population, workplace health promotion measures can be useful to facilitate managers’ mental health resources and support them in their occupational challenges.

Mindfulness in particular may be an effective leader self-development approach to develop capabilities that managers require to handle challenges, people, and change successfully (Hougaard and Carter, 2018; Urrila, 2021). We define a manager as a person holding a managerial or leadership position in a company leading their direct reports (Urrila, 2021). Furthermore, we define upper-level managers as persons in these positions who have high responsibilities and lead lower-level managers. Mindfulness describes a trait, a state, a set of mind-training practices and a multidimensional set of cognitive skills that can be enhanced with practice (Baer and Lykins, 2011). Kabat-Zinn (2003) defines mindfulness as “the awareness that emerges through paying attention on purpose, in the present moment, and nonjudgmentally to the unfolding of experience moment by moment.” Mindfulness interventions typically originate from Buddhist meditation practices (Kabat-Zinn, 2003) and employ a combination of practices such as meditation, psychoeducation, and experiential group training (Kersemaekers et al., 2018). For managers as a specific target group, prior studies suggest mindfulness improves their personal well-being, work performance and leadership quality (Roche et al., 2014; King and Haar, 2017; Urrila, 2021). Furthermore, studies indicate leader mindfulness also benefits their employees, e.g., through improved well-being, job satisfaction, work performance and improvements on an interpersonal level (Verdorfer, 2016; Arendt et al., 2019; Reb et al., 2019; Schuh et al., 2019). On the one hand, mindfulness may improve managers’ capabilities that are not defined as leadership capabilities *per se*, but that nonetheless promote better leadership. Examples include better regulations of managers’ emotions and attention (Hülsheger et al., 2013; Dietl and Reb, 2021), better decision-making and problem-solving (Butler and Gray, 2006). On the other hand, mindfulness may improve leadership-specific capabilities such as leading in complex work environments (Reitz et al., 2020), handling change (Goldman-Schuyler et al., 2017) or ‘post-conventional’ leadership (Baron and Cayer, 2011). In light of the demands of the modern working world, strengthening their own health and capability to perform is essential for managers. In the ICT sector, managers may be particularly exposed to higher ICT demands and an ever-changing work environment (Zeike et al., 2019; Stadin et al., 2021). Thus, facilitating mindfulness of ICT-managers may be particularly important to promote their personal well-being and capabilities.

Despite sound clinical evidence and a growing amount of research on the role of mindfulness in workplace settings, exploring mindfulness in management settings is relatively new (Donaldson-Feilder et al., 2019; Urrila, 2021). So far, there is good evidence that mindfulness-based interventions positively affect mental health and well-being outcomes across various occupational settings. Two meta-analyses of randomized controlled trials demonstrated that workplace mindfulness interventions effectively diminish negative outcomes such as stress, depression, burnout, mental distress, and somatic complaints while promoting positive outcomes such as mindfulness, well-being, compassion, job performance and job satisfaction (Lomas et al., 2019; Vonderlin et al., 2020). For managers in particular, two systematic reviews concluded that mindfulness interventions have the potential to increase managers’ well-being, resilience, and leadership capabilities (Donaldson-Feilder et al., 2019; Urrila, 2021). While the quality and nature of the analyzed interventions varied, advancing the development of leaders through leader-tailored mindfulness trainings was encouraged. Particularly, more follow-up assessments of mindfulness trainings and examinations of work performance effects should be conducted (Vonderlin et al., 2020).

This study’s primary objective is to investigate differences in upper-level managers’ mental health-related outcomes and work performance at the beginning of (*t*0), immediately after (*t*1) and 3 months after (*t*2) a workplace mindfulness training in a German ICT-company. More specifically, we explore differences in managers’ trait mindfulness, psychological well-being, health literacy, and work performance using self-reported measures in an exploratory one-group pre-post design. Furthermore, we explore workplace barriers and facilitators of long-term mindfulness practice and further potential explanation of the training’s effectiveness by embedding qualitative analysis of managers’ open-ended answers. Considering that mindfulness interventions in corporate and management settings are still few, this study can add value by contributing knowledge about the efficacy of a mindfulness training for upper-level managers in an ICT-company setting.

## 2. Theoretical background

Considering the sound evidence for mental health and well-being benefits of mindfulness interventions, we employ a resource-oriented theoretical approach: We assume a direct relation of organizational and personal resources promoted during the training with an increase in managers’ capacities to cope with demands of their personal health and workplace challenges. First, underlying theoretical principles of the training that were specified by the training provider are described. These principles are not empirically investigated in this study, but are described to make the training approaches more understandable. Second, we apply the Job Demands-Resources (JD-R) model to elaborate on the theoretical background for the expected increase in outcome variables.

### 2.1. Training goals and underlying principles

The training ‘Healthy and Mindful Leadership’ was developed and conducted by an external training provider. Accordingly, the training program had three main goals: (1) promoting managers’ understanding as to why strengthening health literacy is necessary in light of the digitalized working; (2) strengthening managers in their function as role models for employees; (3) strengthening managers in their function as health literacy promoters for employees. Mindfulness was a key element within the program. More specifically, establishing mindfulness practice and strengthening mindfulness was considered fundamental for managers’ health literacy, stress management, work performance and health. Aside from mindfulness, another means of achieving the goals was imparting knowledge about the impacts of digitalized work and promoting mindful handling of digital work media.

The providers’ rationale for the training program was based on the health-oriented leadership (HoL)-concept of Franke et al. (2014) and the immunity-to-change approach of Kegan and Lahey (2009). Based on the HoL-concept, ‘healthy leadership’ is a way of leading in which the manager not only focuses on work performance, but also on promoting their own and employees’ health (‘self-care’ and ‘staff-care’). More specifically, managers’ self-care comprises (1) the value the manager attributes to their own health, (2) the level of mindfulness enabling managers to notice when they demand too much of themselves, and (3) the conscious behavior and actions that promote health (Franke et al., 2014).

Mindfulness as a meta-competency is an essential key to healthy leadership, as understood by the training provider. The ‘immunity to change’-approach of Kegan and Lahey (2009) was used as an additional training principle. Accordingly, the provider assumes that mindfulness facilitates ‘personal transformation and creative skills’. More specifically, habituated reactive behavior should be transformed into new, creative behavior to enable healthy leadership and health promotion. The presumption is that mindfulness and self-awareness help with promoting creative behavior. The training provider took up four steps of behavior transformation (Kegan and Lahey, 2009): (1) from reactive unhealthy behavior that the manager is *un*aware of, (2) toward reactive unhealthy behavior that the manager is *aware* of, (3) toward new creative behavior that is only achievable with a high level of awareness and willpower, (4) toward creative behavior that is achievable with a lower level of awareness after the new behavior became a habit. The training provider assumed that entrenched behavior can be noticed, consciously stopped in the moment and be replaced by new behavior through mindfulness and increased self-awareness.

### 2.2. Job demands-resources model

The Job Demands-Resources (JD-R) model by Bakker and Demerouti (2007) is a framework explaining how work-related factors can influence employee well-being, health, and performance. Accordingly, the impacts of work-related factors are explained through two different mechanisms (Bakker et al., 2004): First, a motivational process based on the effect of job resources can help explain outcomes such as work performance. Second, a health impairment process caused by job demands can describe resulting health outcomes such as exhaustion. On the one hand, job characteristics are classified as job demands, which are “physical, social or organizational aspects of the job that require sustained physical or mental effort and are therefore associated with physiological and psychological costs” (Demerouti et al., 2001). On the other hand, job resources “help reach work-related goals, reduce job demands and the associated costs, and stimulate personal growth and development” (Tummers and Bakker, 2021). Job resources enhance efficient coping with work demands and are thus able to weaken the link between job demands and serious health outcomes (Xanthopoulou et al., 2009; Lesener et al., 2019). Aside from organizational job resources, personal resources complete the JD-R model. These personal resources (e.g., resiliency, optimism, and self-efficacy) function as motivators for employees to reach their goals and influence the ability to make use of job resources (Bakker and de Vries, 2021).

A combination of low job resources and high job demands may lead to burnout. Hence, organizations should optimize job characteristics through increasing job resources and improving job demands. Furthermore, Bakker and de Vries (2021) suggest that organizational resources such as healthy leadership and personal resources may support employees in regulating their job strain in an effective way. For example, an organization can provide a training for managers to enhance their personal resources by developing new skills and enable them to cope with job demands (Bakker and Demerouti, 2007). In the context of JD-R theory, leadership can impact job demands, job resources and personal resources in different ways (Tummers and Bakker, 2021). Accordingly, there is an indirect link between leadership and job outcomes *via* job demands and job resources (Schaufeli, 2015). For example, leaders can prioritize work tasks, when employees experience high workload or they may increase job resources by giving employees more autonomy.

We view mindfulness as a personal resource of managers (Grover et al., 2017) and well-being as an outcome of the motivational process according to the JD-R model. Taking previous findings on the impact of mindfulness training on these outcomes into account, we hypothesize:

*Hypothesis 1a* (H1a): The mindfulness training significantly increases managers’ self-reported level of mindfulness from baseline (t0) to post-intervention (t1).

*Hypothesis 1b* (H1b): The mindfulness training significantly increases managers’ self-reported level of mindfulness from baseline (t0) to 3-months follow-up (t2).

*Hypothesis 2a* (H2a): The mindfulness training significantly increases managers’ self-reported level of well-being from baseline (t0) to post-intervention (t1).

*Hypothesis 2b* (H2b): The mindfulness training significantly increases managers’ self-reported level of well-being from baseline (t0) to 3-months follow-up (t2).

Furthermore, we argue that health literacy is a personal resource similar to mindfulness that can support managers in dealing with job strain in an efficient manner (Fiedler et al., 2018). Health literacy can be defined as “the cognitive and social skills which determine the motivation and ability of individuals to gain access to, understand and use information in ways which promote and maintain good health” (Nutbeam, 1998). For managers, health literacy can be considered an important construct due to its’ substantial contribution to well-being and the workplace as a valuable setting for its’ promotion. We argue that if managers know their own limits regarding their health, they can develop healthy and sustainable coping mechanisms that will prevent exhaustion. Since managers are subject to ICT demands, the mindfulness training might promote managers’ health literacy through becoming aware their own behavior and acquiring knowledge about impacts of digitalized work on health. We hypothesize:

*Hypothesis 3a* (H3a): The mindfulness training significantly increases managers’ level of self-reported health literacy from baseline (t0) to post-intervention (t1).

*Hypothesis 3b* (H3b): The mindfulness training significantly increases managers’ level of self-reported health literacy from baseline (t0) to 3-months follow-up (t2).

Lastly, work performance can be regarded as another outcome of the motivational process according to the JD-R model. Accordingly, mindfulness practice can be considered a means to strengthen and manage personal resources better, such as well-being and health literacy, that ultimately affect work performance. Prior research suggests mindfulness affects managers’ work performance positively (Shonin et al., 2014; King and Haar, 2017). Assuming that mindfulness practice is a personal resource affecting managers’ perceived work performance, we hypothesize:

*Hypothesis 4a* (H4a): The mindfulness training significantly increases managers’ level of self-reported work performance from baseline (t0) to post-intervention (t1).

*Hypothesis 4b* (H4b): The mindfulness training significantly increases managers’ level of self-reported work performance from baseline (t0) to 3-months follow-up (t2).

Finally, we assume that the effectiveness of health promotion measures depends on their contextuality which requires exploration (Craig et al., 2018). Contextual aspects in specific industries, such as workplace challenges of upper-level ICT-managers, might affect an intervention’s acceptability and outcome scores, requiring an intervention and evaluation tailored to the targeted group and environment (Glomb et al., 2011; Sutcliffe et al., 2016). Case studies can help explain contextual factors under which participants act and can answer questions of “how” and “why” managers choose to practice mindfulness or not (Yin, 2003). A qualitative approach is useful for investigating the reasons behind certain behavior, beliefs and attitudes of people and providing comprehensive results (Patton, 2002). Thus, qualitative insights can substantiate the quantitative results in this study. This approach adds value by identifying managers’ daily life experiences and actual application of mindfulness trainings at the workplace. By embedding a qualitative approach, we strive to gain more knowledge about two explorative questions regarding the contextual factors and effectiveness of the intervention:

1. What barriers and facilitators in the workplace regarding a sustainable mindfulness practice do managers experience?
2. What other possible effects are reported following the training?

## 3. Materials and methods

### 3.1. Sample and procedure

A mixed-methods approach with a quantitative one-group pre-post design embedding subsequent qualitative analyses was used for this study (Schoonenboom and Johnson, 2017). Outcome measures at the beginning of a workplace mindfulness training (*t*0), immediately after the last half-day group session (*t*1) and 3 months after (*t*2) the mindfulness training were compared. All upper-level managers (approximately 1800) in a large German ICT-company were invited to participate. Participation was limited to a maximum of 12 participants per group with a total of 13 available groups, resulting in 156 managers who were admitted to the training. Thus, not all managers who registered for the training could be admitted. The trainings were conducted in consecutive groups. Participants were recruited using announcements on the company intranet website and announcements of upper-level health managers in executive committee meetings. The managers were told they could participate in a free 2-months mindfulness training during working hours. Participation in the training and in the self-report surveys was voluntary. The authors developed the survey cooperating with three coaches who conducted the training and three upper-level health managers in the ICT-company. The survey included Likert scales (for the outcomes mindfulness, well-being, and health literacy), closed questions with categorial response options, and open-ended questions. Work performance as an outcome was collected using one item. Mandatory questions were not included. Responses to open-ended questions were used for qualitative content analysis. Open-ended questions addressed (1) workplace barriers and (2) workplace facilitators for daily mindfulness practice, (3) positive effects of the training, 4) suggested changes of workplace conditions, and (5) open feedback. The open-ended questions are provided in Supplementary material S1.

Training participants were invited to complete the surveys at the beginning of the training (t0), immediately after the last half-day group session (*t*1) and 3 months after (*t*2) the training. At the start of the study, the surveys were administered both paper-pencil-based (*t*1) and online (*t*0, *t*2) using the web tool LimeSurvey (LimeSurvey GmbH, Hamburg, Germany). After the training switched to a fully digital mode in October 2020, all following surveys (*t*0, *t*1, *t*2) were administered online. Data was collected pseudonymously and matched between time points using a personal code stated by the participant. Aside from the free mindfulness training, no other incentives were offered to the participants. Finally, the training and surveys were conducted consecutively with 13 groups of managers between October 2019 and April 2021. A total of 56 managers (36% of all registered managers) finished the training and all three surveys. Using *t*0 data, mean differences on outcome variables and differences in the distribution of demographics were examined between participants who dropped out after the *t*0 survey and participants who engaged in all surveys (*t*0, *t*1, *t*2). Independent *t*-tests (for continuous variables) and fisher’s exact tests (for remaining demographics) showed no significant differences between the drop-outs (*n* = 54) and completers (*n* = 56). For further quantitative analyses, we decided against using or imputing data of drop-outs due to the large proportion of missing data on outcome variables (>40% for *t*1 and *t*2) (Jakobsen et al., 2017). Thus, for quantitative analyses, we used complete cases only. For qualitative analyses, we used all available open responses (including drop-outs) to enrich findings. Due to the COVID-19 pandemic, the group sessions described in the next section first switched to a hybrid mode (one live and one digital session) in September 2020 and finally to an entirely digital mode in October 2020. Thus, seven groups (*n* = 33) participated in the live on-site training mode, two groups participated in the hybrid mode (*n* = 10) and four groups (*n* = 13) participated in the digital mode of the group sessions.

### 3.2. Intervention: “Healthy and mindful leadership” training

**Figure 1:**
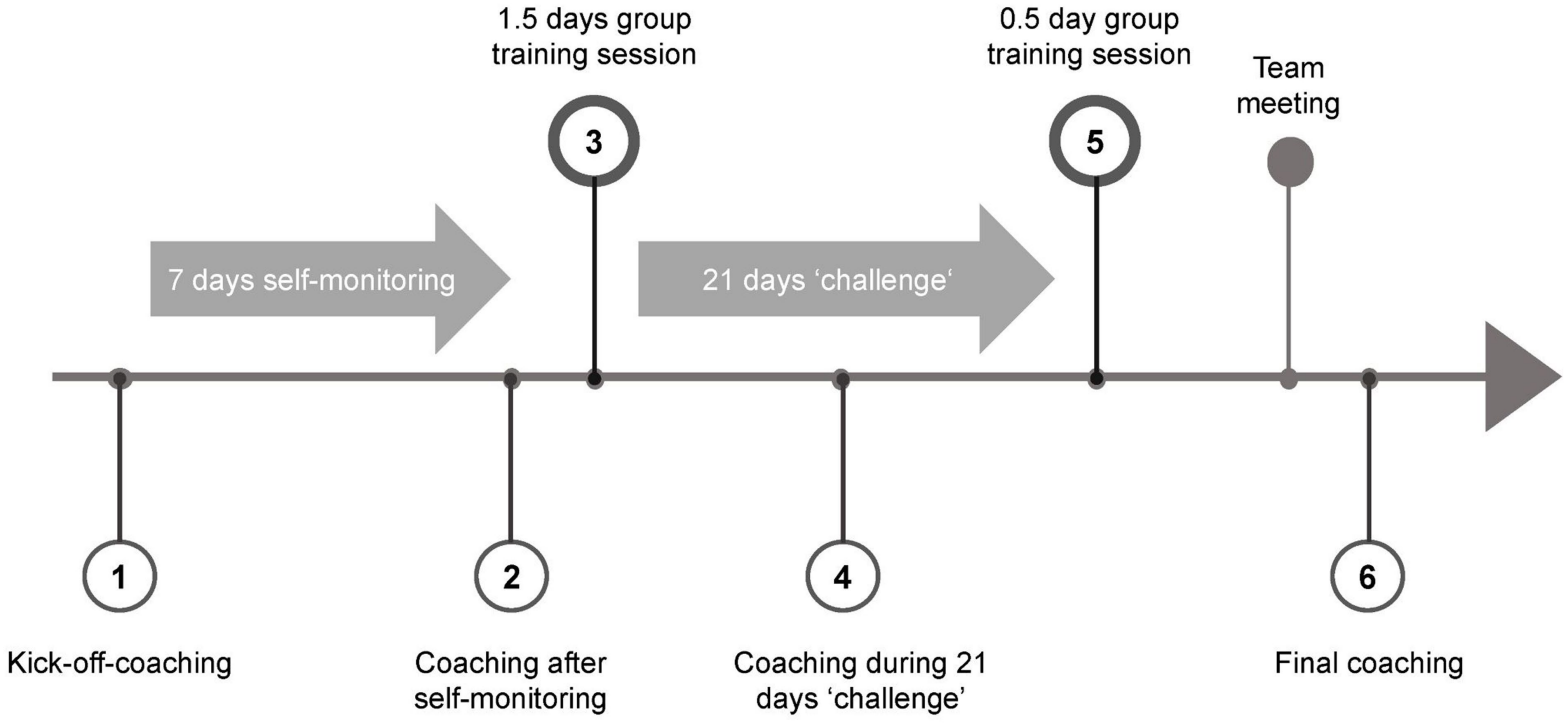
Outline of the mindfulness training program.

The training followed four steps: (1) Raising self-awareness, (2) raising awareness of healthy leadership, (3) self-management through mindfulness, and (4) planning and taking actions. The steps were realized using individual 30 min-coaching sessions *via* video calls, independent practice, and group training sessions (either live on-site, hybrid or digital). Refer to Figure 1 for the outline of the training program. The training was conducted by three coaches who had 10 or more years of experience in coaching and teaching in the field of mindfulness and leadership in international companies. The first step of the training program started with raising self-awareness (1). The program began with a kick-off-coaching between each manager and one of the coaches to clarify aims, benefits, procedures and expectations of the training. In the coaching, managers were instructed to self-monitor their health behavior and digital behavior for 7 days without judging it. This self-observation phase aimed at helping managers become aware of and consciously perceiving their present reactive behavior patterns. The managers and coaches analyzed these observations in a subsequent individual coaching session to facilitate self-awareness and understanding of the manager’s current situation. Participants were also given a physical and digital textbook with an overview and background information of the training program.

Afterwards, 1.5-days group sessions were conducted in which managers were sensitized for the subjects of health, impacts of digitalization, self-management, and managers’ own function as role models. This corresponds with step (2) of the training program: raising awareness of healthy leadership. In the sessions, this included providing information about the increase in sickness absences due to mental disorders, the demands of the digitalization of the working world as both a change and a risk for health, digital stress, managers function as role models in workplace health promotion and the importance of leading oneself and others in a healthy and mindful way. Furthermore, information was provided about the benefit for managers and the scientific evidence about the impact of mindfulness-based breathing and meditation on well-being and performance. Additionally, tools to cope with the impact of digital work, such as information overload, multitasking, and work interruptions, were suggested. This included structuring the working day in a more effective and healthy way and reducing self-interruptions by practicing mindfulness.

The experiential group sessions focused on learning and applying such mindfulness and breathing exercises to help managers establish new behavior. Applying the knowledge and the learned exercises in everyday life afterwards corresponds with step (3) of the training program: self-management through mindfulness. By using the knowledge and mindfulness exercises as tools, managers are supposed to manage their resources better, facilitate calmness and composure, and thus be enabled to lead themselves better in everyday life. In the subsequent ‘21-days challenge,’ participants were tasked with implementing new behavior in their everyday life based on the exercises of the 1.5-days group session and their own behavioral goals. This corresponds with step (4) of the training program: planning and taking actions. For this, managers were instructed to develop an individual action plan for behavioral change in the 1.5-days group session by answering the questions: ‘What will I do? How and why will I do it? What challenges will I face and how will I master them?’ Based on the immunity-to-change approach, managers were taught about challenges of behavior change and provided with tips for establishing new behavior. Here, practicing mindfulness aimed at becoming aware of implicit convictions and thinking patterns keeping the manager from establishing behavioral change. The aim of the ‘21-days-challenge’ was that new, creative behavior, that was initially used consciously, becomes unconscious behavior and a natural part of everyday life in the long term. This ‘challenge’ was supported by a web app and a peer coaching partnership with a colleague from the same group to support implementation of the acquired knowledge and mindfulness practice into everyday life. The web app comprised audio and video tutorials for mindfulness practices learned in the group session, further mindfulness practices, documents from the group session, a self-monitoring diary and a tracking tool for the ‘21-days challenge.’ Additionally, the coaches conducted a third coaching session with each manager during the first half of the challenge. The purpose of the coaching was helping managers detect and overcome obstacles in the challenge and supporting them in reaching their behavioral goals. In subsequent half-day group training sessions, managers analyzed their personal accomplishments and obstacles during the challenge, refreshed their knowledge of mindfulness and breathing exercises, and decided on further behavioral goals and actions. Afterwards, the managers prepared a team meeting with their direct reports to transfer the acquired knowledge and exercises to their team. This corresponds with step (4) of the training program, planning and taking actions, to promote the role of the manager as a disseminator of knowledge and encourage the manager to actively promote health in their team. Finally, a last coaching session was conducted to analyze each manager’s perception of their development in well-being, work performance and health, and to support further plans of behavioral changes.

### 3.3. Measures

#### 3.3.1. Mindfulness

The German version of the Mindfulness Attention Awareness Scale (MAAS, Brown and Ryan, 2003; Michalak et al., 2008) was used for measuring trait mindfulness. The MAAS measures the frequency of mindfulness states or, more specifically, the awareness of and attention to what is happening in the present. All 15 items are phrased negatively (e.g., “I find it difficult to stay focused on what’s happening in the present” or “I rush through activities without being really attentive to them”). Response options ranged from 1 (almost always) to 6 (almost never). Higher values indicate higher levels of mindfulness. The MAAS showed high internal consistency rates (Michalak et al., 2008; Osman et al., 2016). Internal consistency in our sample was Cronbach’s *α* = 0.9 (*t*2).

#### 3.3.2. Well-being

The German version of the World Health Organization Well-Being Index (WHO-5) was used for measuring psychological well-being [World Health Organization (WHO), 1998]. The WHO-5 is a positively phrased 5-item measure assessing psychological well-being within the last 2 weeks. Participants are asked how often they felt cheerful, relaxed, active, well-rested upon waking and interested in things in their daily life. Response options ranged from 0 (at no time) to 5 (all the time). The WHO-5 has been used extensively in international research showing adequate validity and high reliability (Topp et al., 2015; Sischka et al., 2020). Internal consistency in our sample was Cronbach’s *α* = 0.87 (*t*2).

#### 3.3.3. Health literacy

We used a four-point Likert scale based on Lenartz’ (2012) health literacy questionnaire to assess managers’ health literacy. Lenartz’ underlying questionnaire proved reliable and valid with different samples (Lenartz, 2012; Kuhlmann et al., 2015) and displayed adequate internal consistency in a study exploring the health literacy of managers (Fiedler et al., 2018). Based on the questionnaire, we developed a short scale by choosing and adapting six items worded to fit the health literate behavior of managers (e.g., “I have set clear goals for my physical and mental fitness” or “As far as my health is concerned, I am very much in control of myself and I can manage myself effectively”). Response options ranged from 1 (strongly disagree) to 4 (strongly agree). Internal consistency of the scale in our sample was Cronbach’s *α* = 0.82 (*t*2). The scale is provided in Supplementary material S2.

#### 3.3.4. Work performance

After the overall survey was adapted (starting with the fourth training group), the German translation of the World Health Organization Health and Work Performance Questionnaire (HPQ) was added to measure work performance. The HPQ is a self-report questionnaire for measuring “the workplace costs of health problems in terms of reduced job performance, sickness absence, and work-related accidents-injuries” (Kessler et al., 2003). We used the item ‘absolute presenteeism’ as a measure of work performance which is assessed by the following question: “On a scale from 0 to 10, where 0 is the worst job performance anyone could have at your job and 10 is the performance of a top worker, how would you rate your overall job performance on the days you worked during the past 4 weeks?” The question indicates a person’s estimation of their work performance on a scale of 0 (worst performance) to 10 (top performance). The HPQ shows high reliability and validity (Kessler et al., 2004). More specifically, the ‘absolute presenteeism’ measure in the HPQ was considered a valid approach to quantify work performance loss (Scuffham et al., 2014), suggesting adequate test–retest reliability (Kawakami et al., 2020).

#### 3.3.5. Subjective training benefits at follow-up

At 3-months follow-up, participants were asked whether they still applied the exercises learned in the training and, if not, what kept them from still applying the exercises in an open-ended question. After the survey was adapted (starting with the fourth training group), participants were also asked about the subjective benefits of the training using a Likert scale at 3-months follow-up. The scale comprised five items with response options ranging from 1 (strongly disagree) to 4 (strongly agree). Example items are: “The intervention encouraged me to incorporate small moments of mindfulness into my daily life” and “The measure helped me pay more attention to myself and my health.” The internal consistency of the scale was Cronbach’s *α* = 0.76. The scale and an exploratory factor analysis are provided in Supplementary material S2.

#### 3.3.6. Sociodemographic and work-related characteristics

Sociodemographic and work-related characteristics of participants were collected at baseline (*t*0). The following data was collected: upper management level (top, middle, low), managerial experience (in years), weekly overtime hours (<2 h, 2–5 h, >5–10 h, >10 h), gender (female, male, diverse) and age groups (<30, 31–40, 41–50, 51–55, and >55 years). Starting with the fourth training group, items for age groups were adapted for the remaining data collection (<18, 18–24, 25–44, 45–64, and >65 years). Furthermore, managers were asked whether they had already participated in a workplace health promotion measure in the past (yes/no).

### 3.4. Statistical analysis

We used descriptive statistics to report participants’ sociodemographic and work-related characteristics. Assumptions for normality of outcome measures were tested. Depending on the measurement level, correlations were computed to examine associations between outcomes, sociodemographic and work-related variables at *t*0. Data of participants was clustered within six joined training groups based on the chronological proximity of the individual training groups (groups 1 to 3 = cluster 1, groups 4 + 5 = cluster 2, groups 6 + 7 = cluster 3, groups 8 + 9 = cluster 4, groups 10 + 11 = cluster 5, groups 12 + 13 = cluster 6). Kruskal–Wallis-tests were conducted to examine mean differences in outcome variables and managerial experience between training clusters (*t*0, *t*1, *t*2) and group session modes (live on-site, hybrid, digital). Kruskal–Wallis-tests were chosen to account for differing distributions in participant numbers between the clustered training groups. Analyses yielded no significant differences in outcome means. Furthermore, fisher’s exact test (age groups, gender, management level, overtime hours) was conducted to examine differences between training groups for the remaining demographic and work-related variables. There was a significant difference in the management level distribution between training groups. The remaining analyses yielded no significant differences.

We used repeated measures ANOVAs and Bonferroni-adjusted post-hoc analysis to examine within-subject changes in outcome measures across time points (*t*0, *t*1, *t*2). Deviance from sphericity was tested (Mauchly’s sphericity test). Analyses were conducted for matched cases with complete data for all outcome measures. Missing values were not imputed. Due to occasional missing values, the size of the analysis sample in the ANOVAs varied depending on the outcome measure. This resulted in an analysis sample of *n* = 54 for mindfulness and well-being, *n* = 55 for health literacy, and *n* = 41 for work performance. All analyses were performed using SPSS version 28 (IBM, Armonk, NY, United States). Mean scores of outcome measures at the time points (*t*0, *t*1, *t*2), mean differences between time points, *p*-values and effect sizes were estimated. Significance for all analyses was estimated at an alpha of *p* < 0.05. Partial eta squared η^2^ was calculated with η^2^ = 0.01 indicating a small effect, η^2^ = 0.06 indicating a medium effect and η^2^ = 0.14 indicating a large effect (Cohen, 1988).

Furthermore, a series of multiple linear regression analyses was conducted to account for the potential impact of the COVID-19 periods on outcome measures. As management level varied significantly between training groups, we included it in the regression analyses and further controlled for age, gender, and outcome values at *t*0 (for outcomes at *t*1) or *t*1 (for outcomes at *t*2). COVID period, management level, age and gender were coded as dummy variables. For regression analyses, the age groups of the training groups 1–3 and 4–13 were merged. The variable ‘upper management level’ was recoded to exclude one participant reporting an ‘unknown’ management level. Linear regression analyses were conducted for four outcome variables as dependent variables (mindfulness, well-being, health literacy, work performance) with post and follow-up values each (*t*1, *t*2).

Based on times of COVID-19 waves and lockdown phases in Germany (Schilling et al., 2021), three periods were differentiated in which the trainings were conducted: (1) before the outbreak of COVID-19, (2) summer 2020, and (3) winter 2020. The ‘summer plateau’ of 2020 (weeks 21–39) was characterized by mild cases of COVID and substantial loosening of social restrictions established during the first COVID wave. In contrast, the second COVID-19 wave started in fall 2020 and peaked at the end of 2020 (week 40 of 2020 to week 8 of 2021). This phase was characterized by severe cases, and a lockdown with strict social restrictions. Based on the assumed differences in the societal impacts due to the epidemiological outcomes and strictness of social restrictions, we assigned the training participants to one of these three conditions. Three groups finished the training before the outbreak of COVID-19 (1), four groups finished the training during the summer plateau 2020 (2), and six groups finished the training during winter 2020 (3).

### 3.5. Qualitative analysis of open-ended responses

Qualitative methods were used to describe managers’ perspectives of the training and to identify perceived barriers and facilitators for applying the learned techniques regularly after the end of the training. Two female authors, LS and KS, with experience in qualitative analyses conducted qualitative content analysis (Kuckartz, 2010). MaxQDA 2022 (VERBI GmbH, Berlin, Germany) was used to code the data. The first coding round was performed by LS, while KS carried out the second round. After a discussion between the two authors and agreeing on a final coding scheme – confirming the transparency of the coding (Helfferich, 2011)– the categories were applied to all qualitative responses by LS. The example responses were translated into English by both authors. The final coding scheme with definitions and example quotations can be found in Supplementary material S1. The coding scheme comprises deductive main categories derived from open-ended questions in the surveys (*t*0, *t*1, *t*2). Main categories included (1) workplace barriers, and (2) workplace facilitators of regular long-term application of mindfulness exercises and (2) facilitators of transferring the knowledge and exercises learned in the training to followers within managers’ teams. Further main categories comprised (3) suggested changes to managers’ overall workplace conditions to make them more health promoting, and (4) further positive effects of the training that managers perceived. The coding scheme also includes subcategories developed inductively based on the material. This is in line with the qualitative research approach of Kuckartz (2010). Accordingly, this exploratory research is still based on the stated research questions, but allows themes to emerge from the data and reflect participants’ experiences. We followed the guidelines of qualitative reporting criteria (COREQ) by Tong et al. (2007) regarding two applicable domains for the provided open answers: We listed personal characteristics of the authors who conducted qualitative analysis and reported the data analysis and findings in detail.

## 4. Results

### 4.1. Sociodemographic and work-related characteristics

The majority of managers in the sample were male (76.8%) and between 45 and 64 years old (62.5%) or older than 51 years (16.8%) (see Table 1). Most managers were in the middle level (66.1%) of upper-level management, followed by the top level (19.6%) and low level (12.5%). The mean managerial experience was 9.6 years (SD = 6.2). Less than half of managers (46.6%) had already participated in a workplace health promotion measure in the past. More than half of managers (53.6%) reported they worked more than 5 up to 10 h overtime weekly, while a third (30.4%) reported working more than 10 h overtime weekly. The ICT-company’s human resources department compared the distribution of sociodemographic and work-related variables of participants with in-house data and confirmed the distributions were representative for the company’s managerial population.

**Table 1:** Participants’ characteristics at baseline (*n* = 56).

Characteristics	All participants
Gender	*n* (%)
Female	13 (23.2)
Male	43 (76.8)
Age group	
25–44 years (groups 4–13)	7 (12.5)
45–64 years (groups 4–13)	35 (62.5)
41–50 years (groups 1–3)	5 (8.9)
> 51 years (groups 1–3)	9 (16.8)
Upper management level	
Top	11 (19.6)
Middle	37 (66.1)
Low	7 (12.5)
Unknown	1 (1.8)
Managerial experience in years	
Mean (SD)	9.6 (6.2)
Minimum	1
Maximum	28
Previous participation in a workplace health promotion measure	
Yes	26 (46.4)
No	28 (50)
Missing	2 (3.6)
Average hours working overtime per week	
Under 2 h	2 (3.6)
2–5 h	7 (12.5)
More than 5 up to 10 h	30 (53.6)
More than 10 h	17 (30.4)

### 4.2. Mindfulness and well-being

As shown in Table 2, participants had a lower baseline score (*t*0) in mindfulness as measured by the MAAS. The ANOVA indicated a significant improvement of participants’ mindfulness [*F*(2,106) = 3.376, *p* = 0.038, η_p_^2^ = 0.06, *n* = 54]. More specifically, Bonferroni-adjusted post-hoc analysis revealed a significant improvement of mindfulness between baseline and 3-months follow-up. However, a significant improvement was not observed for mindfulness between t0 and t1 although the MAAS score slightly increased between these two time points. The effect size was moderate (η^2^ = 0.060). Additionally, a slight but statistically insignificant increase from *t*1 to *t*2 was observed for managers’ mindfulness (mean difference of 0.1 in the MAAS between *t*1 and *t*2). Furthermore, there was a significant improvement in well-being after the training [*F*(2,106) = 73.019, *p* < 0.001, η_p_^2^ = 0.17, *n* = 54]. *Post hoc* analysis showed a significant improvement in well-being scores between baseline (*t*0) and after the training (*t*1) as well as between baseline (*t*0) and 3-months follow-up (*t*2). The effect size indicated a large effect (η^2^ = 0.170). While there was a decrease in well-being from *t*1 to *t*2, well-being remained higher at both *t*1 and *t*2 compared to *t*0.

**Table 2:** Mean outcome scores at baseline (*t*0), after the training (*t*1), and follow-up (*t*2), mean differences between time points and effect sizes.

Scale (Possible range)	Mean (SD) *t*0	Mean (SD) *t*1	Mean (SD) *t*2	Mean difference t0–t1 (SE)	Cohen’s *d* of pairwise comparison	Mean difference t0–t2 (SE)	Cohen’s *d* of pairwise comparison	Mean difference t1–t2 (SE)	Cohen’s *d* of pairwise comparison	Partial eta-squared of overall ANOVA
Mindfulness (MAAS), (1–6) (*n* = 54)	3.9 (0.72)	4.1 (0.59)	4.2 (0.74)	−0.13 (0.08)	−0.216	−0.209* (0.08)	−0.342	−0.09 (0.08)	−0.141	0.060
Well-Being (WHO-5) (0–25), (*n* = 54)	14.0 (4.37)	16.3 (3.80)	15.4 (4.31)	−2.32*** (0.48)	−0.657	−1.35* (0.52)	−0.380	0.96 (0.49)	0.281	0.170
Health literacy (1–4), (*n* = 55)	2.9 (0.61)	3.1 (0.58)	3.09 (0.53)	−0.20*** (0.05)	−0.521	−0.19** (0.06)	−0.485	0.01 (0.05)	0.024	0.144
Absolute presenteeism (HPQ), (0–10), (*n* = 41)	7.1 (1.41)	7.7 (1.23)	7.7 (0.97)	−0.68** (0.19)	−0.573	−0.61* (0.21)	−0.474	0.07 (0.21)	0.055	0.149

*P*-values were Bonferroni-adjusted for multiple testing of mean differences between time points. *p < 0.05, **p < 0.01, ***p < 0.001.

### 4.3. Health literacy

Compared to baseline values, participants’ health literacy scores significantly improved after the training [*F*(2,108) = 9.067, *p* < 0.001, η_p_^2^ = 0.14, *n* = 55]. *Post hoc* analysis showed a significant improvement of participants’ health literacy when comparing scores at baseline (*t*0) with scores immediately after the training (*t*1). A significant improvement in health literacy was also observed when comparing scores at baseline (*t*1) with scores at 3-months follow-up (*t*2). The effect size indicated a large effect (η^2^ = 0.144). However, health literacy scores at *t*1 and *t*2 were nearly identical.

### 4.4. Work performance

Participants estimated their work performance during the past 4 weeks (absolute presenteeism) to a mean of 7.1 on a scale of 1–10 at baseline (*t*0). There was a significant improvement in perceived work performance after the training [*F*(2.80) = 7.008, *p* = 0.002, η_p_^2^ = 0.15, *n* = 41]. More specifically, *post hoc* analysis showed a significant improvement of perceived work performance when comparing scores at baseline (*t*0) with scores immediately after the training (*t*1) and with scores at 3-months follow-up (*t*2). The effect size indicated a large effect (η^2^ = 0.149). Work performance scores at *t*1 and *t*2 were nearly identical.

### 4.5. Results of correlation and regression analyses

Table 3 presents bivariate correlations between managers’ sociodemographic variables, work-related variables and outcomes at *t*0, *t*1, and *t*2. Using Pearson correlation, analyses show that mindfulness, well-being, health literacy and the item for work performance were significantly associated. The significance of correlations differed across time points. More specifically, managers’ mindfulness and well-being were significantly associated with each other across all time points (*t*0, *t*1, *t*2). Health literacy was only associated with mindfulness at *t*0 and *t*2, but correlated significantly with well-being at all time points. The work performance item (absolute presenteeism) was significantly associated with mindfulness (*t*0, *t*2), well-being (*t*0, *t*1, *t*2), and health literacy (*t*1) in the respective time points. The work performance item at *t*2 also significantly correlated with managerial experience and the management level. Furthermore, Spearman rho showed a significant association between age group and managerial experience. Lastly, chi-square value showed a significant association between management level and training group cluster.

**Table 3:** Associations between variables (correlation coefficients and chi-square values).

	1	2	3	4	5	6	7	8	9	10	11	12	13	14	15	16
(1) Managerial experience (in years)																
(2) Management level	0.02															
(3) Gender	0.05	4.05														
(4) Age group (training groups 4–13)	0.43**	0.02	0.42													
(5) Training group cluster	0.30	28.77*	0.34	4.56												
Outcomes at *t*0																
(6) Mindfulness (MAAS)	0.13	−0.10	0.03	−0.24	0.17											
(7) Well-being (WHO-5)	0.06	−0.10	0.08	0.16	0.33	0.45**										
(8) Health literacy	0.11	−0.05	0.00	0.02	0.19	0.38**	0.37**									
(9) Absolute presenteeism	0.23	0.08	0.23	0.06	0.40	0.34*	0.5**	0.28								
Outcomes at *t*1																
(10) Mindfulness (MAAS)	−0.06	−0.15	0.03	−0.09	0.31	0.62**	0.41**	0.13	0.25							
(11) Well-being (WHO-5)	−0.21	−0.01	0.16	0.10	0.34	0.27*	0.64**	0.22	0.40**	0.36**						
(12) Health literacy	−0.14	−0.04	0.16	0.07	0.32	0.11	0.34*	0.79**	0.17	0.12	0.34*					
(13) Absolute presenteeism	0.08	−0.05	0.06	−0.06	0.35	0.21	0.32*	0.32*	0.60**	0.24	0.21	0.13				
Outcomes at *t*2																
(14) Mindfulness (MAAS)	0.09	0.02	0.02	0.20	0.17	0.66**	0.51**	0.36**	0.31	0.62**	0.43**	0.26	0.10			
(15) Well-being (WHO-5)	−0.03	0.06	0.04	0.01	0.33	0.37**	0.57**	0.12	0.23	0.41**	0.60**	0.20	0.23	0.68**		
(16) Health literacy	−0.05	−0.03	0.13	0.17	0.25	0.24	0.40**	0.75**	0.13	0.09	0.38**	0.76**	0.14	0.45**	0.46**	
(17) Absolute presenteeism	0.44**	0.38*	0.06	0.20	0.46	0.16	0.42**	0.28	0.44**	0.20	0.19	0.25	0.28	0.46**	0.43**	0.23

Correlations between continuous variables (managerial experience, mindfulness, well-being, health literacy and absolute presenteeism) were calculated using Pearson correlation coefficient. Correlations between ordinal variables (age group and management level) were calculated using Spearman rank correlation coefficient (rho). Correlations between categorial variables (gender and training group cluster) were calculated using Pearson contingency coefficient. Correlations between continuous variables and categorial variables were calculated using Eta coefficient (with continuous variables as dependent variables). Correlations between continuous variables and ordinal variables were calculated using Spearman rank correlation coefficient (rho). Associations between categorial and ordinal variables were calculated using Pearson chi-square value. *P < 0.05, **P < 0.01. Significant correlations (two-tailed) are bold.

Furthermore, Table 4 presents a summary of regression analyses with the COVID-19 period as a predictor of mindfulness, well-being, health literacy and work performance. The dummy variables ‘before COVID’ and ‘summer 2020’ were compared to the reference ‘winter 2020’ regarding the impact of the COVID period. Coefficients showed that participants who finished the training before the outbreak of COVID-19 had significantly higher mindfulness at t1 compared to participants who finished the training in winter 2020 [*F*(7,46) = 5.975, *p* < 0.001, with *R*^2^ = 0.476, adjusted *R*^2^ = 0.397]. In contrast, participants who finished the training in summer 2020 had significantly lower well-being at *t*1 compared to the ‘winter 2020’ group [*F*(7,45) = 7.467, *p* < 0.001, with *R*^2^ = 0.537, adjusted *R*^2^ = 0.465], but higher well-being at *t*2 [*F*(7,46) = 6.066, *p* < 0.001, with *R*^2^ = 0.480, adjusted *R*^2^ = 0.401]. Additionally, participants in the high management level had significantly lower well-being at *t*1 compared to middle management level. In contrast, participants in the low management level had significantly higher well-being at *t*2 compared to the middle management level. Due to missing cases, the dummy variable ‘before COVID-19’ was excluded in the models predicting work performance. Participants who finished the training in summer 2020 rated their work performance at *t*1 significantly higher compared to the ‘winter 2020’ group [*F*(6,33) = 6.097, *p* < 0.001, with *R*^2^ = 0.526, adjusted *R*^2^ = 0.439]. With the exception of work performance at *t*2, all regression models were significant (adjusted for *t*0 or *t*1 values, age, gender, and management level). Aside from *t*0 and *t*1 values, no significant associations were observed for mindfulness at *t*2 [*F*(7,45) = 4.874, *p* < 0.001, with *R*^2^ = 0.431, adjusted *R*^2^ = 0.343] or for health literacy at both *t*1 and *t*2 [*t*1: *F*(7,46) = 12.186, *p* < 0.001, with *R*^2^ = 0.650, adjusted *R*^2^ = 0.596; *t*2: *F*(7,46) = 10.551, *p* < 0.001, with *R*^2^ = 0.616, adjusted *R*^2^ = 0.558].

**Table 4:** Summary of linear regression analyses with the COVID-19 period predicting mindfulness, well-being, health literacy, and work performance.

	*T*1					*T*2				
	*B*	SE	*β*	*t*	*p*	B	SE	*β*	*t*	*p*
Mindfulness (MAAS)										
MAAS at *t*0/*t*1	0.51	0.09	0.63	5.67	0.001***	0.83	0.15	0.65	5.45	0.001***
Before COVID-19^a^	0.49	0.17	0.38	2.93	0.005**	−0.13	0.24	−0.08	−0.56	0.58
Summer 2020^a^	0.21	0.14	0.18	1.46	0.15	0.02	0.20	0.01	0.10	0.92
High management level^b^	−0.06	0.18	−0.04	−0.30	0.76	0.01	0.24	0.01	0.05	0.96
Low management level^b^	−0.02	0.19	−0.01	−0.11	0.91	0.31	0.25	0.14	1.23	0.23
Age group: 25–44 years^c^	−0.17	0.16	−0.13	−1.12	0.27	−0.28	0.20	−0.16	−1.36	0.18
Gender: female^d^	−0.02	0.15	−0.01	−0.12	0.91	−0.02	0.20	−0.01	−0.08	0.94
Well-being (WHO-5)										
WHO-5 at *t*0/*t*1	0.51	0.09	0.60	5.90	0.001***	0.82	0.13	0.72	6.10	0.001***
Before COVID-19^a^	0.79	1.02	0.10	0.77	0.45	−0.20	1.24	−0.02	−0.16	0.88
Summer 2020^a^	−2.22	0.90	−0.29	−2.47	0.017*	2.49	1.11	0.28	2.24	0.030*
High management level^b^	−3.02	1.15	−0.32	−2.62	0.012*	1.61	1.45	0.15	1.11	0.27
Low management level^b^	−1.17	1.13	−0.11	−1.04	0.30	3.58	1.38	0.29	2.59	0.013*
Age group: 25–44 years^c^	−0.29	0.95	−0.03	−0.31	0.76	−0.74	1.16	−0.07	−0.64	0.53
Gender: female^d^	−0.78	0.97	−0.09	−0.80	0.43	−0.18	1.13	−0.02	−0.16	0.87
Health literacy										
Health literacy at *t*0/*t*1	0.74	0.09	0.78	8.71	0.001***	0.70	0.09	0.76	8.00	0.001***
Before COVID-19^a^	−0.02	0.14	−0.02	−0.16	0.88	−0.07	0.14	−0.06	−0.54	0.59
Summer 2020^a^	−0.14	0.12	−0.12	−1.17	0.25	0.10	0.11	0.10	0.92	0.36
High management level^b^	0.01	0.15	0.003	0.03	0.98	0.10	0.14	0.08	0.71	0.48
Low management level^b^	−0.01	0.15	−0.008	−0.09	0.93	0.13	0.15	0.09	0.92	0.36
Age group: 25–44 years^c^	0.04	0.12	0.03	0.36	0.72	−0.22	0.12	−0.18	−1.86	0.07
Gender: female^d^	−0.18	0.12	−0.13	−1.42	0.16	−0.06	0.12	−0.05	−0.53	0.60
Absolute presenteeism										
Absolute presenteeism at *t*0/*t*1	0.68	0.12	0.78	5.86	0.001***	0.25	0.12	0.31	2.09	0.04*
Before COVID-19^a^	–	–	–	–	–	–	–	–	–	–
Summer 2020^a^	0.76	0.31	0.31	2.43	0.021*	0.09	0.30	0.05	0.31	0.76
High management level^b^	1.09	0.54	0.27	2.03	0.050	−0.99	0.49	−0.31	−2.02	0.052
Low management level^b^	0.10	0.45	0.03	0.23	0.82	0.28	0.43	0.10	0.64	0.53
Age group: 25–44 years^c^	0.68	0.40	0.21	1.71	0.10	−0.64	0.38	−0.26	−1.71	0.10
Gender: female^d^	0.08	0.36	0.03	0.23	0.82	0.27	0.34	0.12	0.78	0.44

Models adjusted for age, gender, and *t*0 (for *t*1 outcomes) or *t*1 (for *t*2 outcomes). COVID period, management level, age and gender were coded as dummy variables. ^a^Reference category: Winter 2020. ^b^Reference category: Middle management level. ^c^Reference category: Age group 45–64 years. ^d^Reference category: Male. *p < 0.05, **p < 0.01, ***p < 0.001.

### 4.6. Subjective training benefits at follow-up

At 3-months follow-up, 47 out of 56 participants (84%) confirmed they still practiced the mindfulness exercises learned in the training. These participants agreed that they still perceived training benefits at 3-months follow-up by having integrated healthy behavior into everyday life with a mean agreement of 3.3 on a scale of 1–4 (minimum value of 2.6, maximum value of 4.0, *n* = 42).

### 4.7. Qualitative findings

For qualitative analyses, we used all available open responses (including drop-outs). Thus, the qualitative sample contains all of the provided answers regardless of whether participants were excluded in the quantitative analysis due to missing answers. In total, 57 participants answered at least two open-ended questions, while not all of them completed every single question. In sum, 175 questions were content analyzed. Table 5 presents an overview of categories and first level sub-categories. In Supplementary material S1, a more detailed overview of all categories and number of coded answers for each category is provided.

**Table 5:** Overview of categories and first level sub-categories.

Category	Sub-category
Change of work conditions\increasing job resources	Increasing autonomy
	Demarcation between work and leisure
	Fixed workplace and work environment
	Attitude regarding mindfulness
	Retreat spaces
	Regulations
	Role of supervisor
	Sharing of information
	Other
Facilitators of daily mindfulness practice	Contextual factors work environment
	Means and gadgets
	Culture and attitude
	Other
Barriers of daily mindfulness practice	Lack of rooms
	Lack of repetition
	Complexity
	Lack of culture (and acceptance)
	Distributed team
	Lack of prioritization by supervisors
	Time pressure
	Noise level
	Work-related reachability
	High work load
	Nothing
	Lack of time
	Lack of motivation
	Working from home
Positive training effects	More conversation about mindfulness
	More conscious perception
	More reflection
	Acceptance for mindfulness practice increased
	Follow up activities
	Behaviors integrated in everyday life due to mindfulness training
Feedback regarding effectiveness	Increase of well-being
Desire for roll-out	

#### 4.7.1. Barriers of daily mindfulness practice

Reported barriers to daily application of the learned mindfulness exercises involved a perceived lack of follow-up measures or lack of repetition, motivation and energy. One participant stated:

*“[I am] working from home, which means that I sit at the computer early in the morning until late in the evening and have no energy for other topics.”*

Managers mostly stated a lack of a workplace culture, where mindfulness is commonly accepted. Another aspect was the lack of prioritization by supervisors regarding themes such as mindfulness, since daily work routines already filled the day and left no focus and time for mindfulness. One manager stated:

*“I need a visible and clear commitment, at least for our entire department, that mindfulness and health stand above all else. Then the processes will also work.”*

High workload, time pressure, and work-related reachability were also mentioned as barriers to daily mindfulness practice. Additionally, a lack of suitable rooms and the noise level at the workplace prevented managers from daily practice, while other managers reported no barriers exist.

#### 4.7.2. Facilitators of daily mindfulness practice

Managers were asked to name three factors, that would enable them to practice mindfulness daily. Regarding their work environment, managers named budget, autonomy (especially time and breaks) and rooms as necessary requirements. Other factors included a workplace culture that accepts mindfulness and role models who practice mindfulness themselves. This is accompanied by acceptance and support of mindfulness from other colleagues and supervisors:

“*It is also important that - especially the professional - environment practices individual and mutual mindfulness.*”

According to managers’ statements, communicating mindfulness practice to all employees on a broad scale and establishing mindfulness networks could lead to more motivation for practice among staff. Furthermore, personal skills, attitude, learning material and apps were named for facilitating mindfulness practice at the workplace. Other ideas included more training offers in an online or on-site format with a trainer or coach. Four participants expressed their desire for an organizational roll-out of the training. Integration of training practices in daily work routines, training reminders and frequent repetition could be a key component:

*“Without a reminder, current topics will have high potential to eclipse this very positive, but short impulse.”*

#### 4.7.3. Suggested change of workplace conditions

Managers suggested changes to their workplace conditions regarding different topics to make them more health promoting. They mostly named retreats and free space as enablers for a health-promoting workplace. One manager answered:

*“Personally, I don’t see any promotion of mindfulness in terms of spatial arrangements or regulations yet.”*

Furthermore, a positive attitude toward mindfulness in the whole organization and the support of supervisors and management were mentioned. Additional suggestions addressed less workload, less time pressure, a permanent workplace and a strict separation of work and leisure.

#### 4.7.4. Positive training effects at follow-up

Eleven managers openly reported further positive training effects they observed. The responses involved topics such as the effectiveness of breathing and mindfulness practices, integration of healthy behavior into daily life and communication about mindfulness with colleagues:

“*The measure also ensured that I was able to integrate other behaviors into my everyday life using the methods taught, e.g. adjusting my eating habits or sports exercises.*”

“*Conversations about mindfulness and small mindfulness exercises in the team and with peers [were] increased.*”

Furthermore, managers reported to have initiated follow-up activities such as mindfulness exercises together with their team:

“*The handout after the training to report to [our] teams was a good incentive to initiate follow-up activities, e.g. by scheduling […] 5-minute breaks before the next appointment, by practicing guided mindfulness exercises before team meetings together, acceptance for mindfulness exercises has greatly increased.*”

The open responses demonstrate that respondents had an overall positive perception about the workplace mindfulness training. According to these statements, the training increased a more conscious awareness in daily life and the acceptance of mindfulness trainings:

“*[As positive training effects]: More reflection and awareness of the thoughts that occupy me.*”

Moreover, participants commented that the COVID-19 pandemic impacted the positive effects of the mindfulness training in a negative way. The COVID-19 measures lead to increased strain for managers because of social restrictions and a doubled burden if they worked from home and had to take care of their children at the same time.

## 5. Discussion

The present study found significant increases in measures of mindfulness, psychological well-being, health literacy and work performance immediately after and 3 months after a 2-months mindfulness training. However, the limitations of the study design and the potential impact of the COVID-19 pandemic must be considered when interpreting these findings. Aside from the experiential group sessions and surveys that had to switch to a digital format, managers experienced the training before or during different phases of COVID-19. Managers who completed the training before the outbreak of the pandemic had significantly higher mindfulness scores at *t*1 compared to managers who finished the training in winter 2020. The second COVID-19 wave peaked at the end of 2020 and resulted in a lockdown with strict social restrictions in Germany. For the managers participating in the training in winter 2020, the social impacts of the pandemic may have been a distraction, resulting in lower mindfulness after the training. Furthermore, managers who finished the training in summer 2020 had lower well-being at *t*1 but higher well-being at *t*2 compared to the winter 2020 group. A potential reason for the winter group’s lower well-being at *t*2 could be due to the longer experienced lockdown time, while there may have been other confounders for the difference in well-being at *t*1. Additionally, the summer 2020 group rated their work performance better at *t*1 compared to the winter 2020 group. Seasonal differences and fewer social restrictions during summer may have led to a better performance rating of the summer training groups. One subsequent assumption would be that the effects of the training on mindfulness might have turned out stronger without the presence of COVID-19. Some managers also stated a negative impact of COVID-19 in open responses. Still, the increase in mindfulness, psychological well-being, health literacy and work performance in our study aligns with evidence from previous workplace mindfulness intervention studies. A significant increase in mindfulness (MAAS) was only found at 3-months follow-up. Based on significant differences in outcome means between *t*0, *t*1, and *t*2, the exploratory hypotheses can be accepted with the exception of H1a. A possible explanation for this interesting finding is the implied opposite of the Dunning-Kruger effect (Dunning, 2011): After learning what mindfulness is and how to practice it, participants might have underestimated their own abilities immediately after the training. Underestimating one’s own mindfulness skill as a consequence of becoming sensitive toward mindlessness may apply to mindfulness practitioners (Sauer et al., 2015). At 3-months follow-up, managers may have become more confident in their abilities after implementing mindfulness practices into their daily life for a longer amount of time. This finding concurs with long-term studies suggesting that beneficial outcomes of mindfulness interventions are maintained by continuous mindfulness practice (Solhaug et al., 2019; Galante et al., 2021). Another possible explanation for the significant difference in mindfulness at *t*2 (and not at *t*1) is that the MAAS measures trait mindfulness since personality traits take a longer amount of time to change. Furthermore, previous studies did not focus on health literacy as an outcome of mindfulness interventions. Health literacy is considered a precondition for self-care behavior (Bohanny et al., 2013) and some qualitative studies found managers’ self-care improved through mindfulness practice (Lychnell, 2017; Rupprecht et al., 2019). As mindfulness interventions aim at increasing awareness of one’s own thoughts and feelings, the increase in health literacy scores in our sample is not a surprising, but interesting finding.

Regarding the JD-R model, employee strain should be monitored on a continuous basis, since strain depends on the daily combination of job demands and resources. Therefore, supervisors need to provide support and communicate their vision in an ongoing manner (Bakker and de Vries, 2021). We argue that supervisor support and autonomy can be viewed as resources for mindfulness practice. The open-ended answers showed this is crucial since managers wished for their company and supervisors to clearly communicate a commitment to mindfulness. A workplace culture leaving enough autonomy for practicing mindfulness has to be established first so managers can be role models for mindful and health-promoting behavior. Possible spill-over effects to colleagues (Rupprecht et al., 2019), e.g., through implementing joint mindfulness practice into working routines, face barriers that need to be countered. Perceived available time is a well-known pragmatic barrier to engaging in practices such as meditation (Hunt et al., 2020). Such barriers can be considered as job demands since time pressure and workload were mentioned as hindering working conditions. However, managers also mentioned facilitators that can be considered as job resources. In accordance with the JD-R model, job autonomy was named as one of the distinct requirements for daily mindfulness practice. Furthermore, there were suggestions to increase job resources in order to strengthen health promoting workplace conditions such as retreats or support from supervisors and colleagues regarding mindfulness. This idea involves an organizational culture, where mindfulness is commonly accepted.

Investigating managers’ transformative impact areas such as inner growth and relationships as well as interpersonal organizational outcomes such as the working culture and team performance may follow up on the present study (Urrila, 2021). Moreover, research shows a trend toward self-administered, technology-supported mindfulness interventions for busy managers. Albeit this study revealed no significant differences in outcome measures between different group session modes, there is a need to examine the role of continuous technological support embedded into such interventions to support regular mindfulness practice. Regarding practical implications, the qualitative insights show that especially the working conditions and context must be suitable for mindfulness trainings to have a long-lasting effect. The combination of job demands and job resources must allow enough autonomy and freedom to practice mindfulness, while a supporting organizational culture (including the direct leader) can be the common ground for prioritizing health topics and acceptance of mindfulness practice.

Various limitations to this study need to be acknowledged when interpreting the findings. This study used a one-group pre-post design without a control group or randomization. Thus, we cannot derive causal relationships between participation in the training, time effects and changes in the analyzed outcomes. It was important for the managers in charge of the project at the ICT-company to start the trainings in a relatively short amount of time. A control group and randomization could not be implemented due to practical and organizational restraints such as insufficient time for a pre-intervention phase. Therefore, an explorative one-group pre-post design was considered most appropriate for the setting and situation. As participation in the training and the surveys was voluntary, we face a selection bias in the sample. Self-report surveys may yield socially desired responses, thus participants could have been inclined to rate the training outcomes more positively. Drop-outs occurred due to practical obstacles and data availability: The training provider reported that managers, who were originally admitted to the training, dropped out short-term due to sickness or work schedule conflicts. Hence, these managers did not participate in certain training components or data gathering. Missing data may also have resulted from managers who participated in the training but did not fill out the survey, even though coaches emphasized the importance of responding to the surveys. Making participation in such trainings a higher priority in managers’ schedules may prevent future drop-outs. Nonetheless, analysis of matched cases across a long amount of time can be considered a strength of this study. While the training was conducted in one ICT company and generalizing the findings to different branches may be limited, the investigated company was fairly large and participants worked at various departments. Additionally, according to the company’s human resources department, the distribution of sociodemographic and work-related variables of participants was representative for the company’s managerial population. Still, we face the problem that training participants could generally have a higher health awareness compared to non-participating managers (Ludwig et al., 2020).

The analyses were of an exploratory nature. Due to missing data for work performance and a lacking validation of the health literacy and subjective training benefits scales, the statistical findings should be interpreted with caution. Nonetheless, the present study suggests intrapersonal outcome changes in the essential impact areas of individual leadership capacity. Furthermore, our approach combining quantitative findings with ICT-managers qualitative answers adds value with insights on the effectiveness of mindfulness trainings at the workplace.

While it is possible that coaches could have influenced training outcomes, they did not use the developed surveys as guidance for coaching and training. Rather, we assume the coaches had a professional interest in ensuring that managers reach their training goals, reflect upon their individual experiences, and develop a plan for sustainable behavioral change afterwards. As the data collection at *t*1 took place after the last half-day experiential group session, the temporal proximity might have influenced the reported outcomes. However, data analysis was conducted independent of the coaches and a potential influence of coaches’ actions on training outcomes is more unlikely at data collection 3 months after the training. Due to the study design, we could not discern the effect of specific elements of the training. Future studies could investigate how certain components of mindfulness interventions affect and match the measured outcomes (e.g., items of the MAAS). Still, the outcomes suggest that the applied combination of training elements may have been effective in the training program (Lacerenza et al., 2017). This includes a needs analysis (i.e., clarification of managers’ expectations toward the training in the kick-off coaching), personal feedback in the group sessions, in the subsequent coaching and in peer partnerships, spaced group sessions that took place twice, and multiple delivery methods (e.g., personal conversation, text book, web app). Despite the change of group sessions to a digital format during the COVID-19 pandemic, the overall training structure remained the same. Embedding coaching, learning media such as apps, and peer support within such a structured training program can facilitate transfer and behavioral impact in managers’ daily life, which is supported by the qualitative findings. Transfer into daily life is particularly important since managers have high work demands that compete or interfere with mindfulness practice and habituation of healthy behavior.

## 6. Conclusion

Our exploratory findings suggest the mindfulness training may improve mindfulness, psychological well-being, health literacy and work performance among upper-level ICT-managers. In contrast to the other outcomes, a significant increase in mindfulness was found only at follow-up. Managers who finished the training before the outbreak of COVID-19 had a higher mindfulness score at *t*1 compared to those who finished the training in winter 2020. Qualitative findings suggested managers perceived the integration of mindfulness into daily life as a positive effect following the training. Workplace-related barriers and facilitators for the subsequent daily application of learned mindfulness practices emerged from the findings. In subsequent studies, the shortcomings of the present study should be improved by applying a randomized controlled design, uniform validated scales, and larger samples from different organizations. Collecting employee ratings on managers’ behavior and accounting for mechanisms between outcome variables in analyses could generate more rigorous findings. On a practical level, the responsibility and high workload coming with a managerial position substantiates promoting managers’ self-development capabilities through participation in mindfulness trainings. Conducting such trainings is crucial since managers act as role models and can have a substantial positive impact on employees and organizations.

## Data availability statement

The datasets presented in this article are not readily available because the ICT-company did not agree to make the data available in an accessible repository as the data sets contain sensitive company data. Requests to access the datasets should be directed to KS, kristina.schubin@uni-koeln.de.

## Ethics statement

The study involving human participants was reviewed and approved by the Ethics Committee of the Medical Faculty of the University of Cologne (project identification code: 19-1476). The participants provided their written informed consent to participate in this study.

## Author contributions

KS, HP, and SZ designed and planned the study. SZ and KS collected the data and conducted the statistical analyses. LS and KS conducted the qualitative analyses. KS drafted the manuscript. KS and LS edited the manuscript. SZ, HP, and LS provided substantive suggestions for revisions. All authors contributed to the manuscript, and reviewed and approved the final manuscript.

## Funding

The authors declare that this secondary data analysis study received funding from vivalue GmbH and the University of Cologne. For the purpose of this article, the secondary data analysis, data interpretation and writing were financed by own funds of the University of Cologne. The research group of KS, LS, and HP is funded by the University of Cologne. We acknowledge support for the Article Processing Charge from the DFG (German Research Foundation, 491454339).

## Conflict of interest

SZ was employed by vivalue GmbH. HP is a shareholder of vivalue GmbH. The authors declare that this secondary data analysis study received funding from vivalue GmbH. vivalue GmbH was involved in the development of the study design, data collection and conduction of the initial pragmatic evaluation. vivalue GmbH was not involved in the interpretation of data for this article, the writing, or the decision to submit it for publication.

The remaining authors declare that the research was conducted in the absence of any commercial or financial relationships that could be construed as a potential conflict of interest.

## Publisher’s note

All claims expressed in this article are solely those of the authors and do not necessarily represent those of their affiliated organizations, or those of the publisher, the editors and the reviewers. Any product that may be evaluated in this article, or claim that may be made by its manufacturer, is not guaranteed or endorsed by the publisher.

## References

[ref1] Arendt J. F. W. Pircher Verdorfer A. Kugler K. G. (2019). Mindfulness and leadership: communication as a behavioral correlate of leader mindfulness and its effect on follower satisfaction. Front. Psychol. 10:667. doi: 10.3389/fpsyg.2019.00667, PMID: 30984078 PMC6450257

[ref2] Baer R. A. Lykins E. L. B. (2011). “Mindfulness and positive psychological functioning” in Designing Positive Psychology. eds. Sheldon K. M. Sheldon K. M. Kashdan T. Steger M. F. (New York, NY: Oxford University Press), 335–348.

[ref3] Bakker A. B. de Vries J. D. (2021). Job demands-resources theory and self-regulation: new explanations and remedies for job burnout. Anxiety Stress Coping 34, 1–21. doi: 10.1080/10615806.2020.1797695, PMID: 32856957

[ref4] Bakker A. B. Demerouti E. (2007). The job demands-resources model: state of the art. J. Manag. Psych 22, 309–328. doi: 10.1108/02683940710733115

[ref5] Bakker A. B. Demerouti E. Verbeke W. (2004). Using the job demands-resources model to predict burnout and performance. Hum. Resour. Manag. 43, 83–104. doi: 10.1002/hrm.20004

[ref80] Baron C. Cayer M. (2011). Fostering post‐conventional consciousness in leaders: why and how?. Journal of Mgmt Development 30, 344–365. doi: 10.1108/02621711111126828

[ref6] Bohanny W. Wu S.-F. V. Liu C.-Y. Yeh S.-H. Tsay S.-L. Wang T.-J. (2013). Health literacy, self-efficacy, and self-care behaviors in patients with type 2 diabetes mellitus. J. Am. Assoc. Nurse Pract. 25, 495–502. doi: 10.1111/1745-7599.12017 24170654

[ref7] Brown K. W. Ryan R. M. (2003). The benefits of being present: mindfulness and its role in psychological well-being. J. Pers. Soc. Psychol. 84, 822–848. doi: 10.1037/0022-3514.84.4.822 12703651

[ref8] Butler B. S. Gray P. H. (2006). Reliability, mindfulness, and information systems. MIS Q. 30, 211–224. doi: 10.2307/25148728

[ref9] Cohen J. (1988). Statistical Power Analysis for the Behavioral Sciences. 2nd. Hillsdale, NJ: Lawrence Erlbaum Associates.

[ref10] Craig P. Di Ruggiero E. Frohlich K. L. Mykhalovskiy E. White M. Campbell R.. (2018). Taking Account of Context in Population Health Intervention Research: Guidance for Producers, Users and Funders of Research. Southampton: NIHR Evaluation, Trials and Studies.

[ref11] Demerouti E. Bakker A. B. Nachreiner F. Schaufeli W. B. (2001). The job demands-resources model of burnout. J. Appl. Psychol. 86, 499–512. doi: 10.1037/0021-9010.86.3.499 11419809

[ref12] Dietl E. Reb J. (2021). A self-regulation model of leader authenticity based on mindful self-regulated attention and political skill. Hum. Relat. 74, 473–501. doi: 10.1177/0018726719888260

[ref13] Donaldson-Feilder E. Lewis R. Yarker J. (2019). What outcomes have mindfulness and meditation interventions for managers and leaders achieved? A systematic review. Eur. J. Work Organ. Psy. 28, 11–29. doi: 10.1080/1359432X.2018.1542379

[ref14] Dunning D. (2011). “The Dunning–Kruger effect” in Advances in Experimental Social Psychology. eds. Olson J. M. Zanna M. P.. 1st ed (Amsterdam, Boston, London, New York, Oxford, Paris: Academic Press an imprint of Elsevier), 247–296.

[ref15] Eurofound (2017). Sixth European Working Conditions Survey: Overview Report (2017 Update). Luxembourg: Publications Office of the European Union.

[ref16] Fiedler S. Pfaff H. Soellner R. Pförtner T.-K. (2018). Exploring the association between health literacy and psychological well-being among industry managers in Germany. J. Occup. Environ. Med. 60, 743–753. doi: 10.1097/JOM.0000000000001324, PMID: 29557837

[ref17] Franke F. Felfe J. Pundt A. (2014). The impact of health-oriented leadership on follower health: development and test of a new instrument measuring health-promoting leadership. German J. Hum. Resour. Manage. 28, 139–161. doi: 10.1177/239700221402800108

[ref18] Galante J. Stochl J. Dufour G. Vainre M. Wagner A. P. Jones P. B. (2021). Effectiveness of providing university students with a mindfulness-based intervention to increase resilience to stress: 1-year follow-up of a pragmatic randomised controlled trial. J. Epidemiol. Community Health 75, jech-2020-214390–jech-2020-214160. doi: 10.1136/jech-2020-214390, PMID: 32913130 PMC7116569

[ref19] Glomb T. M. Duffy M. K. Bono J. E. Yang T. (2011). “Mindfulness at work” in Research in Personnel and Human Resources Management. eds. Martocchio J. J. Liao H. Joshi A., vol. 30 (Bradford: Emerald Group Publishing Limited), 115–157.

[ref20] Goldman-Schuyler K. Skjei S. Sanzgiri J. Koskela V. (2017). “Moments of waking up”: a doorway to mindfulness and presence. J. Manag. Inq. 26, 86–100. doi: 10.1177/1056492616665171

[ref21] Grover S. L. Teo S. T. T. Pick D. Roche M. (2017). Mindfulness as a personal resource to reduce work stress in the job demands-resources model. Stress Health 33, 426–436. doi: 10.1002/smi.2726, PMID: 27862960

[ref22] Harms P. D. Credé M. Tynan M. Leon M. Jeung W. (2017). Leadership and stress: a meta-analytic review. Leadersh. Q. 28, 178–194. doi: 10.1016/j.leaqua.2016.10.006

[ref23] Helfferich C. (2011). Die Qualität qualitativer Daten: Manual für die Durchführung qualitativer interviews [The Quality of Qualitative Data: Manual for Conducting Qualitative Interviews]. 4. Auflage. Wiesbaden: VS Verlag für Sozialwissenschaften.

[ref24] Hirschle A. L. T. Gondim S. M. G. (2020). Stress and well-being at work: a literature review. Cienc. Saude Coletiva 25, 2721–2736. doi: 10.1590/1413-81232020257.27902017, PMID: 32667554

[ref25] Hougaard R. Carter J. (2018). The Mind of the Leader: How to Lead Yourself, Your People, and Your Organization for Extraordinary Results. Boston: Harvard Business Review Press.

[ref26] Hülsheger U. R. Alberts H. J. E. M. Feinholdt A. Lang J. W. B. (2013). Benefits of mindfulness at work: the role of mindfulness in emotion regulation, emotional exhaustion, and job satisfaction. J. Appl. Psychol. 98, 310–325. doi: 10.1037/a0031313, PMID: 23276118

[ref27] Hunt C. A. Hoffman M. A. Mohr J. J. Williams A.-L. (2020). Assessing perceived barriers to meditation: the determinants of meditation practice inventory-revised (DMPI-R). Mindfulness 11, 1139–1149. doi: 10.1007/s12671-020-01308-7, PMID: 33664878 PMC7929263

[ref85] Jakobsen J. C. Gluud C. Wetterslev J. J. Winkel P. (2017). When and how should multiple imputation be used for handling missing data in randomised clinical trials - a practical guide with flowcharts. BMC Med Res Methodol 17, 162. doi: 10.1186/s12874-017-0442-1, PMID: 29207961 PMC5717805

[ref28] Kabat-Zinn J. (2003). Mindfulness-based interventions in context: past, present, and future. Clin. Psychol. Sci. Pract. 10, 144–156. doi: 10.1093/clipsy.bpg016

[ref29] Kawakami N. Inoue A. Tsuchiya M. Watanabe K. Imamura K. Iida M.. (2020). Construct validity and test-retest reliability of the world mental health Japan version of the World Health Organization health and work performance questionnaire short version: a preliminary study. Ind. Health 58, 375–387. doi: 10.2486/indhealth.2019-0090, PMID: 32173661 PMC7417506

[ref30] Kegan R. Lahey L. L. (2009). Immunity to change: How to overcome it and unlock the potential in yourself and your organization. Boston: Harvard Business Review Press.

[ref31] Kersemaekers W. Rupprecht S. Wittmann M. Tamdjidi C. Falke P. Donders R.. (2018). A workplace mindfulness intervention may be associated with improved psychological well-being and productivity. A preliminary field study in a company setting. Front. Psychol. 9:195. doi: 10.3389/fpsyg.2018.00195, PMID: 29541039 PMC5836057

[ref32] Kessler R. C. Ames M. Hymel P. A. Loeppke R. McKenas D. K. Richling D. E.. (2004). Using the World Health Organization health and work performance questionnaire (HPQ) to evaluate the indirect workplace costs of illness. J. Occup. Environ. Med. 46, S23–S37. doi: 10.1097/01.jom.0000126683.75201.c5, PMID: 15194893

[ref33] Kessler R. C. Barber C. Beck A. Berglund P. Cleary P. D. McKenas D.. (2003). The World Health Organization health and work performance questionnaire (HPQ). J. Occup. Environ. Med. 45, 156–174. doi: 10.1097/01.jom.0000052967.43131.51 12625231

[ref34] King E. Haar J. M. (2017). Mindfulness and job performance: a study of Australian leaders. Asia Pac. J. Hum. Resour. 55, 298–319. doi: 10.1111/1744-7941.12143

[ref35] Kuckartz U. (2010). Einführung in die computergestützte analyse qualitativer Daten [introduction to computer-assisted analysis of qualitative data], 3 aktualisierte Aufl. Wiesbaden: VS Verl. für Sozialwiss

[ref36] Kuhlmann K. Beauducel A. Predel G. Preuß M. Preuß P. Rudinger G. (2015). Evaluation des Gesundheitsverhaltens Studierender: Gesundheitsbezogene Kompetenzen als Grundlage individueller interventions- und Präventionsmaßnahmen [evaluation of the health behavior in students: health competencies: Foundation for Health Intervention and Prevention]. Diagnostica 61, 163–171. doi: 10.1026/0012-1924/a000143

[ref37] Lacerenza C. N. Reyes D. L. Marlow S. L. Joseph D. L. Salas E. (2017). Leadership training design, delivery, and implementation: a meta-analysis. J. Appl. Psychol. 102, 1686–1718. doi: 10.1037/apl0000241, PMID: 28749153

[ref38] Lenartz N. (2012). Gesundheitskompetenz und Selbstregulation [Health Literacy and Self-Regulation]. Göttingen: V&R unipress University Press.

[ref39] Lesener T. Gusy B. Wolter C. (2019). The job demands-resources model: a meta-analytic review of longitudinal studies. Work Stress 33, 76–103. doi: 10.1080/02678373.2018.1529065

[ref40] Lomas T. Medina J. C. Ivtzan I. Rupprecht S. Eiroa-Orosa F. J. (2019). Mindfulness-based interventions in the workplace: an inclusive systematic review and meta-analysis of their impact upon wellbeing. J. Posit. Psychol. 14, 625–640. doi: 10.1080/17439760.2018.1519588

[ref41] Ludwig S. Starker A. Hermann S. Jordan S. (2020). Inanspruchnahme von Maßnahmen der betrieblichen Gesundheitsförderung in Deutschland – Ergebnisse der Studie gesundheit in Deutschland aktuell (GEDA 2014/2015-EHIS) [the use of workplace health promotion interventions in Germany – results of the study "German health update" (GEDA 2014/2015-EHIS)]. Bundesgesundheitsbl 63, 1491–1501. doi: 10.1007/s00103-020-03239-z, PMID: 33146760 PMC7686008

[ref42] Lychnell L. (2017). When work becomes meditation: how managers use work as a tool for personal growth. J. Manage. Spiritual. Relig. 14, 255–275. doi: 10.1080/14766086.2017.1307782

[ref43] Michalak J. Heidenreich T. Ströhle G. Nachtigall C. (2008). Die deutsche version der mindful attention and awareness scale (MAAS) Psychometrische Befunde zu einem Achtsamkeitsfragebogen [German version of the mindful attention and awareness scale (MAAS) – psychometric features of a mindfulness questionnaire]. Z. Klin. Psychol. Psychother. 37, 200–208. doi: 10.1026/1616-3443.37.3.200

[ref44] Nutbeam D. (1998). Health promotion glossary. Health Promot. Int. 13, 349–364. doi: 10.1093/heapro/13.4.349

[ref45] Osman A. Lamis D. A. Bagge C. L. Freedenthal S. Barnes S. M. (2016). The mindful attention awareness scale: further examination of dimensionality, reliability, and concurrent validity estimates. J. Pers. Assess. 98, 189–199. doi: 10.1080/00223891.2015.1095761, PMID: 26560259

[ref46] Patton M. Q. (2002). Qualitative Research & Evaluation Methods. 3rd. Thousand Oaks, CA: SAGE.

[ref47] Reb J. Chaturvedi S. Narayanan J. Kudesia R. S. (2019). Leader mindfulness and employee performance: a sequential mediation model of LMX quality, interpersonal justice, and employee stress. J. Bus. Ethics 160, 745–763. doi: 10.1007/s10551-018-3927-x

[ref48] Reitz M. Waller L. Chaskalson M. Olivier S. Rupprecht S. (2020). Developing leaders through mindfulness practice. J. Manag. Dev. 39, 223–239. doi: 10.1108/JMD-09-2018-0264

[ref49] Roche M. Haar J. M. Luthans F. (2014). The role of mindfulness and psychological capital on the well-being of leaders. J. Occup. Health Psychol. 19, 476–489. doi: 10.1037/a0037183, PMID: 24933594

[ref50] Rodriguez A. Rodriguez Y. (2015). Metaphors for today’s leadership: VUCA world, millennial and “cloud leaders”. J. Manag. Dev. 34, 854–866. doi: 10.1108/JMD-09-2013-0110

[ref51] Rupprecht S. Falke P. Kohls N. Tamdjidi C. Wittmann M. Kersemaekers W. (2019). Mindful leader development: how leaders experience the effects of mindfulness training on leader capabilities. Front. Psychol. 10:1081. doi: 10.3389/fpsyg.2019.01081, PMID: 31156509 PMC6529524

[ref52] Sauer S. Lemke J. Zinn W. Buettner R. Kohls N. (2015). Mindful in a random forest: assessing the validity of mindfulness items using random forests methods. Personal. Individ. Differ. 81, 117–123. doi: 10.1016/j.paid.2014.09.011

[ref53] Schaufeli W. B. (2015). Engaging leadership in the job demands-resources model. Career Dev. Int. 20, 446–463. doi: 10.1108/CDI-02-2015-0025

[ref54] Schilling J. Tolksdorf K. Marquis A. Faber M. Pfoch T. Buda S.. (2021). Die verschiedenen Phasen der COVID-19-Pandemie in Deutschland: Eine deskriptive analyse von Januar 2020 bis Februar 2021 [the different periods of COVID-19 in Germany: a descriptive analysis from January 2020 to February 2021]. Bundesgesundheitsbl 64, 1093–1106. doi: 10.1007/s00103-021-03394-x, PMID: 34374798 PMC8353925

[ref55] Schoonenboom J. Johnson R. B. (2017). How to construct a mixed methods research design. Kolner Z Soz Sozialpsychol 69, 107–131. doi: 10.1007/s11577-017-0454-1, PMID: 28989188 PMC5602001

[ref56] Schuh S. C. Zheng M. X. Xin K. R. Fernandez J. A. (2019). The interpersonal benefits of leader mindfulness: a serial mediation model linking leader mindfulness, leader procedural justice enactment, and employee exhaustion and performance. J. Bus. Ethics 156, 1007–1025. doi: 10.1007/s10551-017-3610-7

[ref57] Scuffham P. A. Vecchio N. Whiteford H. A. (2014). Exploring the validity of HPQ-based presenteeism measures to estimate productivity losses in the health and education sectors. Med Decis. Making 34, 127–137. doi: 10.1177/0272989X13497996, PMID: 23913916

[ref90] Shonin E. van Gordon W. Dunn T. J. Singh N. N. Griffiths M. D. (2014). Meditation Awareness Training (MAT) for Work-related Wellbeing and Job Performance: A Randomised Controlled Trial. Int J Ment Health Addiction 12:806–823. doi: 10.1007/s11469-014-9513-2

[ref58] Sischka P. E. Costa A. P. Steffgen G. Schmidt A. F. (2020). The WHO-5 well-being index – validation based on item response theory and the analysis of measurement invariance across 35 countries. J. Affect. Disord. Rep. 1:100020. doi: 10.1016/j.jadr.2020.100020

[ref59] Sohail M. Rehmann C. (2015). Stress and health at the workplace – a review of the literature. J. Bus. Stud. Q. 6, 94–121.

[ref60] Solhaug I. de Vibe M. Friborg O. Sørlie T. Tyssen R. Bjørndal A.. (2019). Long-term mental health effects of mindfulness training: a 4-year follow-up study. Mindfulness 10, 1661–1672. doi: 10.1007/s12671-019-01100-2

[ref61] Stadin M. Nordin M. Broström A. Magnusson Hanson L. L. Westerlund H. Fransson E. I. (2021). Technostress operationalised as information and communication technology (ICT) demands among managers and other occupational groups – results from the Swedish longitudinal occupational survey of health (SLOSH). Comput. Hum. Behav. 114:106486. doi: 10.1016/j.chb.2020.106486

[ref62] Sutcliffe K. M. Vogus T. J. Dane E. (2016). Mindfulness in organizations: a cross-level review. Annu. Rev. Organ. Psychol. Organ. Behav. 3, 55–81. doi: 10.1146/annurev-orgpsych-041015-062531

[ref63] Tong A. Sainsbury P. Craig J. (2007). Consolidated criteria for reporting qualitative research (COREQ): a 32-item checklist for interviews and focus groups. Int. J. Qual. Health Care 19, 349–357. doi: 10.1093/intqhc/mzm042, PMID: 17872937

[ref64] Topp C. W. Østergaard S. D. Søndergaard S. Bech P. (2015). The WHO-5 well-being index: a systematic review of the literature. Psychother. Psychosom. 84, 167–176. doi: 10.1159/000376585, PMID: 25831962

[ref65] Tummers L. G. Bakker A. B. (2021). Leadership and job demands-resources theory: a systematic review. Front. Psychol. 12:722080. doi: 10.3389/fpsyg.2021.722080, PMID: 34659034 PMC8514935

[ref66] Urrila L. I. (2021). From personal wellbeing to relationships: a systematic review on the impact of mindfulness interventions and practices on leaders. Hum. Resour. Manag. Rev. 32:100837. doi: 10.1016/j.hrmr.2021.100837

[ref67] Verdorfer A. P. (2016). Examining mindfulness and its relations to humility, motivation to lead, and actual servant leadership behaviors. Mindfulness 7, 950–961. doi: 10.1007/s12671-016-0534-8

[ref68] Vonderlin R. Biermann M. Bohus M. Lyssenko L. (2020). Mindfulness-based programs in the workplace: a meta-analysis of randomized controlled trials. Mindfulness 11, 1579–1598. doi: 10.1007/s12671-020-01328-3

[ref69] Westerman G. Bonnet D. McAfee A. (2015). Leading Digital: Turning Technology into Business Transformation. Boston: Harvard Business Review Press.

[ref70] World Health Organization (WHO) (1998). Wellbeing Measures in Primary Health Care/the Depcare Project. Copenhagen: WHO Regional Office for Europe.

[ref71] Xanthopoulou D. Bakker A. B. Demerouti E. Schaufeli W. B. (2009). Reciprocal relationships between job resources, personal resources, and work engagement. J. Vocat. Behav. 74, 235–244. doi: 10.1016/j.jvb.2008.11.003

[ref72] Yin R. K. (2003). Case Study Research: Design and Methods. 3rd Thousand Oaks, CA: SAGE.

[ref73] Zeike S. Choi K.-E. Lindert L. Pfaff H. (2019). Managers' well-being in the digital era: is it associated with perceived choice overload and pressure from digitalization? An exploratory study. Int. J. Environ. Res. Public Health 16:1746. doi: 10.3390/ijerph16101746, PMID: 31108843 PMC6572357

