# Supplementary material for: A workplace mindfulness training program may affect mindfulness, well-being, health literacy and work performance of upper-level ICT-managers: An exploratory study in times of the COVID-19 pandemic
Source: Front Psychol. 2023 Apr 20;14:994959. doi: 10.3389/fpsyg.2023.994959 (PMC10158731; doi:10.3389/fpsyg.2023.994959)
Supplement: Supplementary file 1 [file Data_Sheet_1.pdf]

Supplementary Material 2: Additional information on scales

A Workplace Mindfulness Training Program May Affect Mindfulness, Well-Being, Health Literacy and Work Performance of Upper-Level ICT-Managers: An Exploratory Study in Times of the COVID-19 Pandemic

Kristina Schubin*, Laura Seinsche, Holger Pfaff and Sabrina Zeike
*** Correspondence:** Kristina Schubin: kristina.schubin@uni-koeln.de

TABLE 1 | ‘Health Literacy’ scale.

**Items**	**Response options**
I take care of my health, even if it costs money, time and personal energy.	1 = Strongly disagree<br>2 = Somewhat disagree<br>3 = Somewhat agree<br>4 = Strongly agree
I plan my life so that I stay healthy.	
I know exactly how to maintain my health and stay up to date on this through books and various media.	
I set clear goals for my physical and mental fitness.	
I have made it a habit to maintain my health and live healthy.	
As far as my health is concerned, I am very much in control of myself and can manage myself effectively.	

TABLE 2 | ‘Subjective Training Benefits’ scale.

**Items**	**Response options**
The intervention enabled me to sustainably integrate more mindfulness into my daily life.	1 = Strongly disagree<br>2 = Somewhat disagree<br>3 = Somewhat agree<br>4 = Strongly agree
The intervention helped me to pay more attention to myself and my health.	
The intervention increased my awareness of my health and mindfulness.	
The intervention encouraged me to incorporate small moments of mindfulness into my daily life.	
The intervention encouraged me to take occasional breaks to foster my mindfulness.	

TABLE 3 | Exploratory Factor Analysis of the Items of the Subjective Training Benefits scale.

**Items**	**Factor 1 loading**
The intervention enabled me to sustainably integrate more mindfulness into my daily life.	.724
The intervention helped me to pay more attention to myself and my health.	.842
The intervention increased my awareness of my health and mindfulness.	.745
The intervention encouraged me to incorporate small moments of mindfulness into my daily life.	.699
The intervention encouraged me to take occasional breaks to foster my mindfulness.	.603

Extraction method: Principal components analysis; Varimax rotation could not be conducted since only one component was extracted.
Factor eigenvalue: 2.639. The factor explained 52,8% of variance in variables.
The scree plot suggested a one factor solution.
